# DNA Methylation-Mediated GPX4 Transcriptional Repression and Osteoblast Ferroptosis Promote Titanium Particle-Induced Osteolysis

**DOI:** 10.34133/research.0457

**Published:** 2024-08-19

**Authors:** Jian Dong, Binjia Ruan, Lijun Zhang, Ai Wei, Chuling Li, Neng Tang, Linxi Zhu, Qing Jiang, Wangsen Cao

**Affiliations:** ^1^ State Key Laboratory of Pharmaceutical Biotechnology, Branch of National Clinical Research Center for Orthopedics, Sports Medicine and Rehabilitation, Division of Sports Medicine and Adult Reconstructive Surgery, Department of Orthopedic Surgery, Nanjing Drum Tower Hospital, Affiliated Hospital of Medical School, Nanjing University, Nanjing, China.; ^2^ Nanjing University Medical School, JiangsuKey Lab of Molecular Medicine, Nanjing, China.; ^3^ Yancheng Medical Research Center, Yancheng First People’s Hospital, Affiliated Hospital of Nanjing University Medical School, Yancheng, China.; ^4^ Yangzhou Precision Research Institute of Kidney Disease, Department of Nephrology, Northern Jiangsu People’s Hospital, Yangzhou, China.

## Abstract

Metal wear particles generated by the movement of joint prostheses inevitably lead to aseptic osteolytic damage and ultimately prosthesis loosening, which are aggravated by various types of regulated cell death of bone. Nevertheless, the exact cellular nature and regulatory network underlying osteoferroptosis are poorly understood. Here, we report that titanium particles (TP) induced severe peri-implant osteolysis and ferroptotic changes with concomitant transcriptional repression of a key anti-ferroptosis factor, GPX4, in a mouse model of calvarial osteolysis. GPX4 repression was accompanied by an increase in DNA methyltransferases (DNMTs) 1/3a/3b and hypermethylation of the *Gpx4* promoter, which were partly mediated by the transcriptional regulator/co-repressor KLF5 and NCoR. Conversely, treatment with SGI-1027, a DNMT-specific inhibitor, resulted in marked reversal of *Gpx4* promoter hypermethylation and GPX4 repression, as well as improvement in ferroptotic osteolysis to a similar extent as with a ferroptosis inhibitor, liproxstatin-1. This suggests that epigenetic GPX4 repression and ferroptosis caused by the increase of DNMT1/3a/3b have a causal influence on TP-induced osteolysis. In cultured primary osteoblasts and osteoclasts, GPX4 repression and ferroptotic changes were observed primarily in osteoblasts that were alleviated by SGI-1027 in a GPX4 inactivation-sensitive manner. Furthermore, we developed a mouse strain with *Gpx4* haplodeficiency in osteoblasts (*Gpx4*^Ob+/−^) that exhibited worsened ferroptotic osteolysis in control and TP-treated calvaria and largely abolished the anti-ferroptosis and osteoprotective effects of SGI-1027. Taken together, our results demonstrate that DNMT1/3a/3b elevation, resulting GPX4 repression, and osteoblastic ferroptosis form a critical epigenetic pathway that significantly contributes to TP-induced osteolysis, and that targeting DNMT aberration and the associated osteoferroptosis could be a potential strategy to prevent or slow down prosthesis-related osteolytic complications.

## Introduction

Periprosthetic osteolysis (PPO) stands as the primary cause of aseptic prosthesis loosening and revision surgery in total joint arthroplasty [1]. It accounts for more than 10% of arthroplasty cases, with morbidity on the rise and a lack of effective therapies [2,3]. Because of repetitive movements, prosthetic implants of various compositions generate wear particles that inadvertently cause a local inflammatory response, overproduction of reactive oxygen species (ROS), and deregulated oxidative stress. As a results, periprosthetic bone resorption occurs, leading to prosthesis failure [4]. As a dynamic tissue, bone undergoes constant regeneration, balanced by proper osteoclastogenesis and osteoblastogenesis. Osteoclasts (OCs) negatively, whereas osteoblasts (OBs) positively, regulate bone biosynthesis to maintain bone homeostasis and proper remodeling [5–9]. PPO induced by metal wear particles is known to be promoted by various types of regulated bone cell death, including necroptosis, apoptosis, pyroptosis, or ferroptosis [10,11]. However, the exact role, cellular property, and regulatory networks controlling ferroptosis in this context are largely unknown.

Ferroptosis is a unique and non-apoptotic type of regulated cell death mainly due to impaired iron metabolism and excessive lipid peroxides [12]. It serves a pivotal function in cancer and neurodegenerative and cardiovascular diseases and is emerging as a crucial player in metal wear particle-induced bone diseases [13–15]. While various pathological events and signaling pathways can influence ferroptosis, increasing studies demonstrate that its regulation is directly controlled by the GSH (glutathione)/GPX4 (glutathione peroxidase 4) signaling pathway. GSH functions as both a free radical neutralizer and a coenzyme of GPX4. Inadequate GSH synthesis leads to reduced production of GPX4, an essential anti-ferroptosis factor that can transform harmful phospholipid hydroperoxides to harmless phospholipid alcohols, thereby preventing ferroptosis [16]. GPX4 deficiency, whether resulting from enzymatic inactivation or degradation, is indicative of ferroptosis. On the other hand, its transcriptional repression could involve both epigenetic and non-epigenetic mechanisms [17,18]. Of particular note, the *Gpx4* promoter contains a typical and conserved CpG island around the transcription starting site, indicating that GPX4 transcription is subject to regulation by epigenetic DNA methylation.

DNA methylation is a common and reversible epigenetic modification that affects over 60% of gene transcriptions during various pathophysiological processes [19,20]. It arises through the action of the DNA methyltransferases DNMT1, DNMT3a, and DNMT3b and is antagonized by the methylcytosine dioxygenases TET1, TET2, and TET3 (ten-eleven translocation). DNMTs add a methyl group (CH_3_-) to the cytosine of CpG dinucleotides, which are enriched as CpG islands in gene promoters and enhancers. These methylated sites attract methyl-binding proteins, transcriptional repressors, and cofactors, leading to the repression of transcription in downstream genes [21]. Suppression of GPX4 caused by DNA methylation-associated metabolism has been reported in ferroptosis of nucleus pulposus cells and synoviocytes, which is due to defective metabolism of homocysteine and glycine [22,23]. However, the molecular pathways leading to this suppression and their functional relevance to metal particle-induced osteolytic ferroptosis remain unclear.

We have previously shown in a well-established mouse model of calvarial osteolysis [1,8] that wear particles of both titanium and cobalt–chromium–molybdenum, the common components of metal prostheses, induce similar peri-implant osteolytic damage [24]. In this study, we investigated the potential impacts of aberrant DNA methylation on titanium particle (TP)-induced ferroptosis. We observed significant GPX4 suppression and osteoblastic ferroptosis, accompanied by *Gpx4* promoter hypermethylation and increases in DNMT1/3a/3b. We then developed a mouse strain of *Gpx4* deficiency in OBs (*Gpx4*^Ob+/−^) and conducted the study using both genetic and pharmacological approaches. Our results demonstrate that DNMT aberration-induced GPX4 suppression and osteoblastic ferroptosis play a crucial role in TP-induced osteolysis and shed light on the clinical treatment of wear particle-induced periprosthetic complications.

## Results

### TPs induce significant GPX4 repression and ferroptosis in a mouse model of calvarial osteolysis

**Fig. 1.**
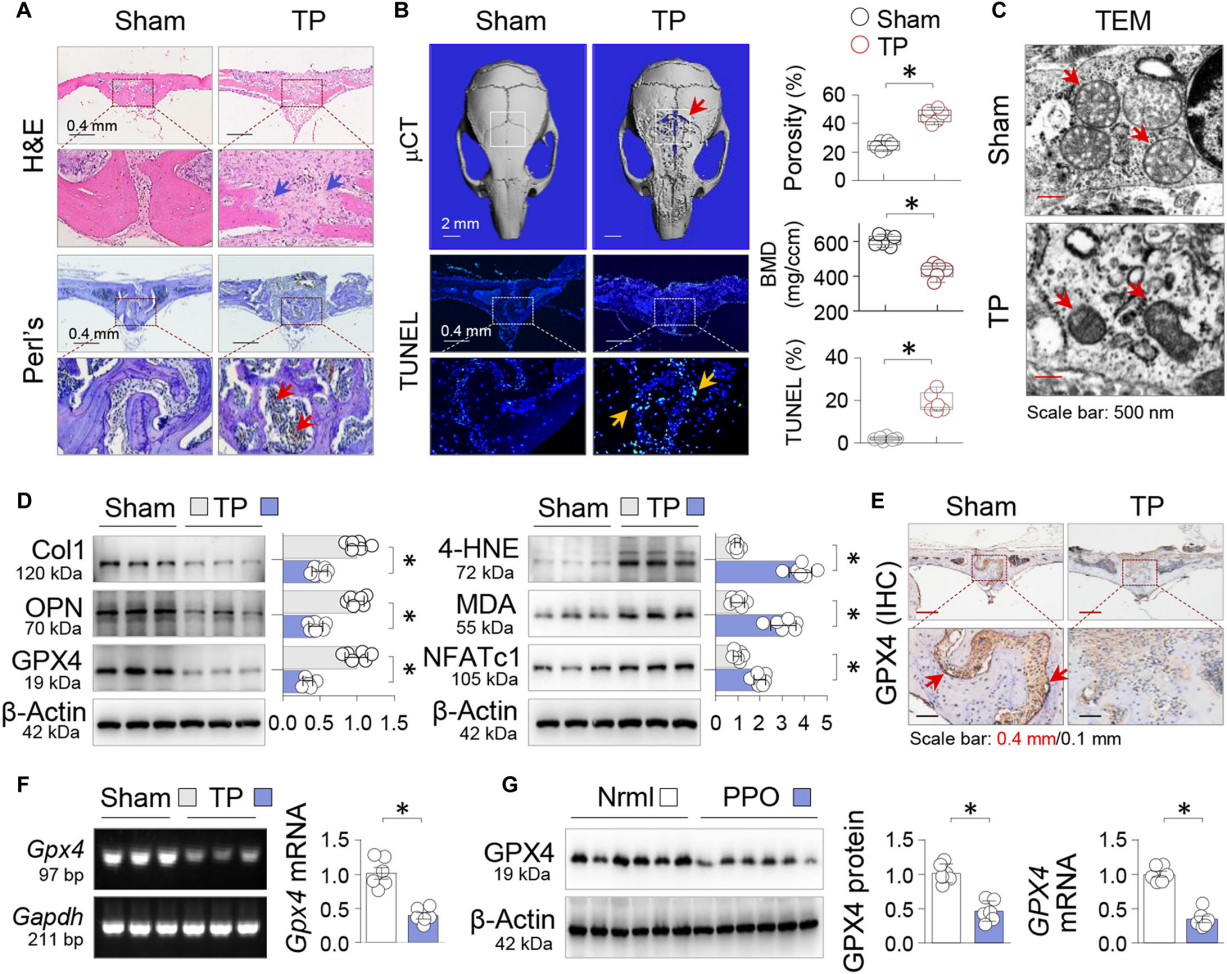
Titanium particles (TPs) induce significant GPX4 repression and ferroptosis in a mouse model of calvarial osteolysis. In a mouse model of calvarial osteolysis, mice underwent Sham or TP treatment for 2 weeks (TP, 20 mg per mouse, *n* = 6). (A) Representative calvarial sections stained by H&E (upper part) or Perls’ prussian blue with DAB enhancement (lower part). Bone resorption and iron deposition were indicated by arrows. (B) Representative 3D micro-x-ray computed tomography (μCT) images of calvaria (upper part) and TUNEL-stained calvarial sections (lower part). Region of interest (ROI) and positively stained cells were indicated by squares and arrows, respectively. Quantitative analyses of bone porosity, bone mineral density (BMD), and TUNEL-positive cell ratio were presented as box-and-whisker plots. *P < 0.05, Student’s *t* test. (C) Representative TEM microphotographs. Normal and ferroptotic mitochondria in TP-treated calvaria were pointed by arrows. (D) Western blotting. Calvarial tissues were examined for Col1, OPN, GPX4, 4-HNE, MDA, and NFATc1, with β-actin serving as control. Three random samples from each group were shown. Quantification was presented as mean ± SEM; *n* = 6; *P < 0.05, Student’s *t* test. (E) Representative calvarial sections stained by immunohistochemistry (IHC) for GPX4. Positively stained cells were pointed by arrows. (F) RT-PCR. *Gpx4* mRNAs from mouse calvaria were assessed by RT-PCR and analyzed on agarose gel. *Gapdh* served as control. Three random samples from each group were shown. Quantification was presented as mean ± SEM. *n* = 6. (G) Perioprosthetic bone tissues from patients receiving joint replacement and normal controls were assayed for GPX4 protein by Western blotting and GPX4 mRNA by qRT-PCR, with β-actin and GAPDH serving as controls, respectively. Quantifications were presented as mean ± SEM. *n* = 6; *P < 0.05, Student’s *t* test.

To gain insights into TP-induced osteolytic ferroptosis, we adopted a well-established mouse model of calvarial osteolysis [1]. After 2 weeks of TP treatment, mice exhibited significant calvarial erosions (Fig. 1A, upper panel), elevated bone porosity (45.6 ± 1.9% versus 24.5 ± 1.2% of control, *P* < 0.05; Fig. 1B, right upper area), and reduced bone mineral density (BMD; Fig. 1B, right lower area). In addition, there was notable iron deposition (Fig. 1A, lower panel) and an increase in positive cells (18.9 ± 1.9% versus 2.1 ± 0.4% of control, *P* < 0.05; Fig. 1B, lower panel) as detected by TUNEL (terminal deoxynucleotidyl transferase–mediated deoxyuridine triphosphate nick end labeling) assay, a validated technique for identifying apoptotic and ferroptotic cells [25]. Transmission electron microscopy (TEM) showed characteristic ferroptotic mitochondrial changes, such as smaller mitochondria and reduced mitochondrial crista after TP treatment (Fig. 1C). TP exposure also resulted in adverse changes in OB and OC markers: an increase in the OC marker NFATc1 (nuclear factor of activated T cells c1) and a decrease in OB markers OPN (osteopontin) and Col1 (type 1 collagen) (Fig. 1D). At the same time, GPX4 was significantly suppressed, accompanied by an increase in lipid peroxidation indicators MDA (malondialdehyde) and 4-HNE (4-hydroxynonenal) (Fig. 1D) [26]. Immunohistochemical staining of calvarial sections showed that GPX4, which was abundant in control tissues, was markedly reduced after TP treatment (Fig. 1E). This reduction was also evident at the mRNA level (Fig. 1F). Examination of clinical samples revealed that GPX4 protein and mRNA levels were high in control bone tissues but significantly decreased in tissues from patients with confirmed prosthesis loosening (Fig. 1G). These results suggest that TP-induced osteolysis may be causally related to GPX4 suppression and associated ferroptosis.

### TP-induced GPX4 suppression coincides with DNMT elevation and *Gpx4* promoter hypermethylation

**Fig. 2.**
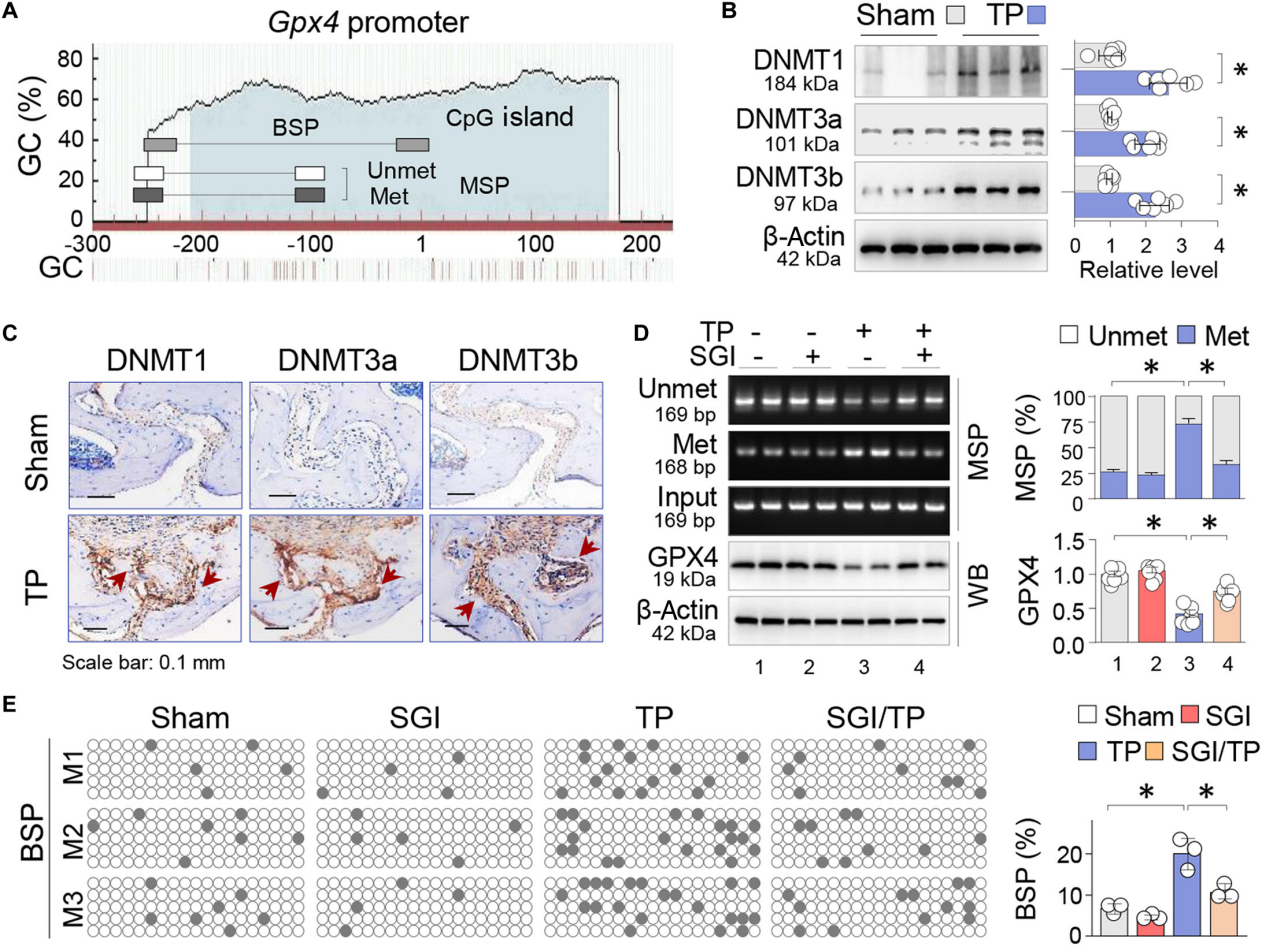
TP-induced GPX4 suppression coincides with DNMT elevation and *Gpx4* promoter hypermethylation. (A) Schematic diagrams of the mouse *Gpx4* promoter. The positions of CpG island and MSP/BSP primers were depicted relative to the transcription starting site. (B) Western blotting of the calvarial tissues from Sham and TP-treated (20 mg per mouse, 2 weeks) mice for DNMT1, DNMT3a, and DNMT3b, with β-actin serving as control. Three random samples per group were shown. Quantification was presented as mean ± SEM; *n* = 6; *P < 0.05, Student’s *t* test. (C) IHC. Representative calvarial sections stained by IHC for DNMT1, DNMT3a, and DNMT3b. The positively stained cells were pointed by arrows. (D) MSP assay and Western blotting. Calvarial tissues from Sham or TP-treated mice with or without SGI-1027 (2.5 mg/kg daily, *n* = 6) were assessed by MSP to assess methylation levels of the *Gpx4* promoter. PCR products were examined via agarose gel electrophoresis. Two random samples from each group were shown. The methylated and unmethylated PCR products were adjusted with input PCR, and quantification was presented as mean ratio ± SEM. The same tissues were assessed for GPX4 protein, and quantification was presented as mean ± SEM. *P < 0.05, 2-way ANOVA. (E) BSP assay of the *Gpx4* promoter. BSP was performed on 3 random animals (designated M1, M2, and M3) from each group. Five cloned PCR products from each animal were sequenced. Each box represents one mouse, and each row of dots in each box represents one cloned sequence, with each dot representing one CpG site. Empty circles denote unmethylated CpGs, whereas black dots represent methylated CpGs. Quantitative data were presented as mean ratio ± SEM of methylated/unmethylated CpGs in total CpGs within the cloned fragments. *P < 0.05, 2-way ANOVA.

To investigate the connection between GPX4 repression and DNA methylation, we performed GPX4 promoter methylation analysis using MetPrimer (http://www.urogene.org/methprimer) and identified a dense CpG island at the −211/170 locus near the transcription starting site (Fig. 2A). This finding indicates that *Gpx4* is sensitive to DNA methylation modification [16,17]. Further examination of DNA methylation-regulating enzymes in calvarial tissues revealed that the enzymatically active DNA methyltransferases DNMT1, DNMT3a, and DNMT3b were all up-regulated following TP treatment (Fig. 2B). IHC staining showed that DNMT1/3a/3b had similar distributions to GPX4 in normal calvaria (Fig. 2C).

We then examined the level of DNA methylation within the Gpx4 promoter by MSP [methylation-specific polymerase chain reaction (PCR)] and observed that TP treatment increased the level of methylation at the −247/−79 locus from 26.5 ± 1.9% to 73.4 ± 4.4% (*P* < 0.05; Fig. 2D). Nevertheless, administration of SGI-1027, a quinoline-based DNMT inhibitor, reduced the level to 33.7 ± 3.3% (*P* < 0.05) and reversed GPX4 suppression (Fig. 2D). To validate the MSP results, we performed a bisulfate-specific PCR (BSP) assay, the gold standard for assessing DNA methylation, which allows testing of each individual CpG site. We randomly tested 3 mice from every group and sequenced 5 cloned PCR products from each animal. The analyzed *Gpx4* promoter region with 19 CpG sites (−248/5) also showed increased methylation from 6.7 ± 0.7% to 20.0 ± 2.2% (*P* < 0.05); however, SGI-1027 treatments reduced the values to 10.5 ± 1.1% (*P* < 0.05; Fig. 2E). These findings suggest that the up-regulated DNMT1/3a/3b are important upstream regulators that cause GPX4 promoter hypermethylation and transcriptional repression.

### DNMT aberration-induced GPX4 suppression is co-regulated by KLF5

**Fig. 3.**
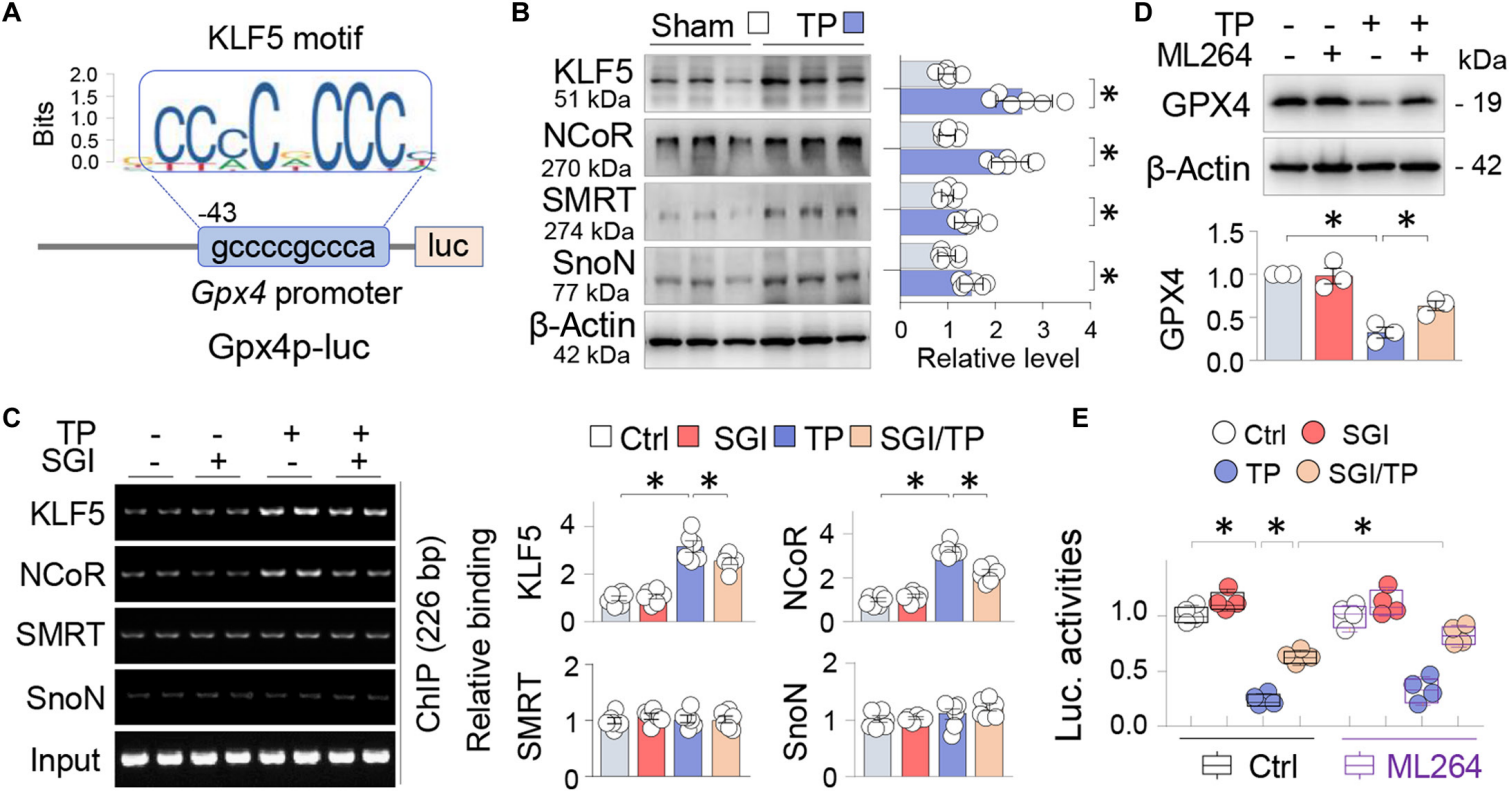
DNMT aberration-induced GPX4 suppression is co-regulated by KLF5. (A) Schematic diagram depicting mouse *Gpx4* promoter luciferase reporter, *Gpx4*p-luc, the KLF5 binding motif, and its position relative to the transcription starting site. (B) Western blot. Calvarial tissues treated with control and TP (20 mg per mouse, 2 weeks) were assayed for KLF5, NCoR, SnoN, and SMRT, with β-actin serving as control. Two random samples per group were shown. Quantification beside blots was presented as mean ± SEM; *n* = 6; *P < 0.05, Student’s *t* test. (C) ChIP. Sham or TP-treated calvaria treated with or without SGI-1027 (2.5 mg/kg daily) were immunoprecipitated first with antibodies to KLF5, SMRT, NCoR, or SnoN, and then the immunoprecipitated DNA fragments were amplified through PCR with primer set specific for the KLF5 motif-containing region of the *Gpx4* promoter, respectively. The non-immunoprecipitated DNA served as control (Input). PCR products were resolved on agarose gels. Quantifications on the right side were presented as mean ± SEM; *n* = 6; *P < 0.05, 2-way ANOVA. (D) Western blot. Primary osteoblasts (OBs) treated without or with TP (100 μg/ml) and the KLF5 inhibitor ML264 (10 μM) for 48 h were assayed for GPX4, with β-actin serving as control. Quantification below was presented as mean ± SD of 3 replicated experiments. *P < 0.05, 2-way ANOVA. (E) Luciferase assay. Primary OBs were transfected with a mouse *Gpx4* promoter-luciferase reporter (*Gpx4*p-luc) and a Renilla luciferase reporter control, and then treated without or with TP (100 μg/ml) and SGI-1027 (10 μM) in the absence or presence of ML264 (10 μM) for 48 h. Luciferase activities of the *Gpx4* promoter reporter were adjusted to Renilla luciferase activities and presented as box-and-whisker plots of 4 independent experiments. *P < 0.05, 3-way ANOVA.

To delve deeper into the regulatory network that controls *Gpx4* transcriptional repression, we examined the *Gpx4* promoter [500 base pairs (bp)] using JASPAR (https://jaspar.genereg.net/) and noticed several binding motifs with varying binding scores for transcription factors such as NRF2 (9.5852), SP1 (11.9437), C/EBPβ (4.0259), and NF-1C (8.5204). Notably, a Kruppel-like factor 5 (KLF5) motif with higher binding score (−43/gccccgccca, 15.465838) was found near the transcription starting site (Fig. 3A). KLF5, a zinc-finger transcriptional factor, possesses both transcription activation and repression capabilities [27]. While KLF5 is acknowledged to hold a significant role in various cancers [28], its role in bone diseases remains largely unexplored.

We found that expression of KLF5 together with the transcriptional co-repressors NCoR (nuclear receptor corepressor), SMRT (silencing mediator of retinoid and thyroid hormone receptor), and SnoN (ski-related novel gene N) increased in TP-treated calvaria (Fig. 3B). A chromatin immunoprecipitation (ChIP) assay further demonstrated that KLF5 and NCoR, but not SMRT or SnoN, bound inducibly to the proximal *Gpx4* promoter containing the KLF5 motifs after TP treatment, which were substantially blocked by SGI-1027 (Fig. 3C). Furthermore, TP-induced GPX4 suppression in primary OBs was partially inhibited by ML264, a KLF5-selective inhibitor (Fig. 3D). Consistent with these results, a luciferase assay using a *Gpx4* promoter/luciferase reporter showed that the transcriptional suppression of the *Gpx4* promoter induced by TP was notably reversed by SGI-1027, and this effect was even more prominently mitigated upon administration of ML264 (Fig. 3E). Collectively, these results suggest that the transcriptional repression of GPX4 induced by TP is co-regulated by KLF5 and NCoR.

### DNMT inhibition by SGI-1027 alleviates TP-incurred GPX4 suppression and ferroptotic osteolysis

**Fig. 4.**
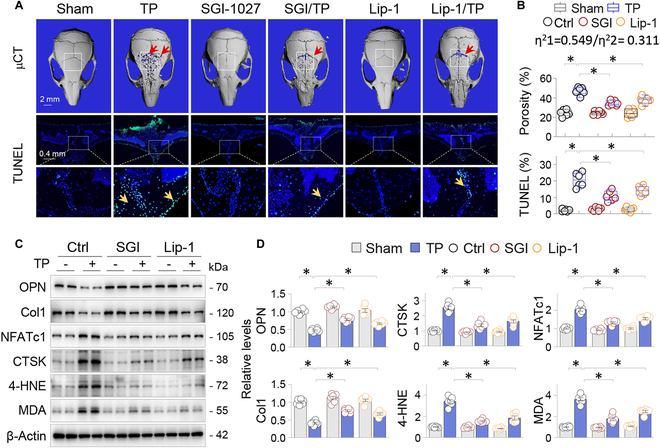
DNMT inhibition by SGI-1027 alleviates TP-incurred GPX4 suppression and ferroptotic osteolysis. Mice were allocated to Sham or TP groups (20 mg per mouse, 2 weeks) and treated with control vehicle (Ctrl), SGI-1027 (2.5 mg/kg daily), or Lip-1 (10 mg/kg daily) (*n* = 6). (A) Exemplary 3D μCT calvarial images and TUNEL-stained calvarial sections. The ROI within μCT images was indicated by squares, while bone resorption areas and TUNEL-positive cells are pointed by arrows. (B) Quantitation of bone porosity and the ratio of TUNEL-positive cells. Data were presented as box-and-whisker plots. *P < 0.05, 2-way ANOVA. The effect size of the interaction between TP-induced calvarial porosity and SGI-1027 (η^2^1) or Lip-1 (η^2^2) intervention was indicated. (C) Western blotting of 2 random calvarial samples from each group for OPN, Col1, CTSK, NFATc1, 4-HNE, and MDA, with β-actin serving as control. (D) Quantification of (C). Data were depicted as mean ± SEM; *n* = 6; *P < 0.05, 2-way ANOVA.

To examine the functional relevance of GPX4 repression and its restoration by DNMT inhibition in vivo, we analyzed the calvaria of Sham and TP-treated mice in the presence or absence of SGI-1027 or liproxstatin-1 (Lip-1), a specific ferroptosis inhibitor that inhibits lipid peroxidation and ferroptotic processes without affecting other types of cell death [25]. The results showed that neither SGI-1027 nor Lip-1 affected the normal calvarial microstructure or the number of TUNEL-positive cells. However, both significantly mitigated the TP-induced adverse changes of calvarial porosity (Fig. 4A and B; 47.2 ± 1.7% of TP versus 34.5 ± 1.1% of SGI-1027/TP and 37.7 ± 1.6% of Lip-1/TP, *P* < 0.05; Fig. 4A and B, upper panels) and reduced the number of TUNEL-positive cells (20.6 ± 1.8% of TP versus 10.7 ± 1.3% of SGI-1027/TP and 14.1 ± 1.1% of Lip-1/TP, *P* < 0.05; Fig. 4A and B, lower panels). We further calculated the effect size (η^2^) of the interaction between TP-induced calvarial porosity and SGI-1027 or Lip-1 intervention. Both SGI-1027 (η^2^1 = 0.549) and Lip-1 (η^2^2 = 0.311) effectively alleviated osteoporotic damage, with SGI-1027 having a stronger capacity (Fig. 4B, inset). Furthermore, TP-induced abnormal expressions of osteoblastic OPN and Col1, osteoclastic NFATc1 and CTSK, and the lipid peroxidation markers 4-HNE and MDA were effectively reversed by SGI-1027 and Lip-1 (Fig. 4C and D). Since Lip-1 specifically targets ferroptosis, these results indicate that GPX4 repression caused by DNMT aberration plays a decisive role in TP-induced ferroptosis and the resulting osteolysis.

### TP-induced GPX4 repression and ferroptotic alterations occur primarily in OBs

**Fig. 5.**
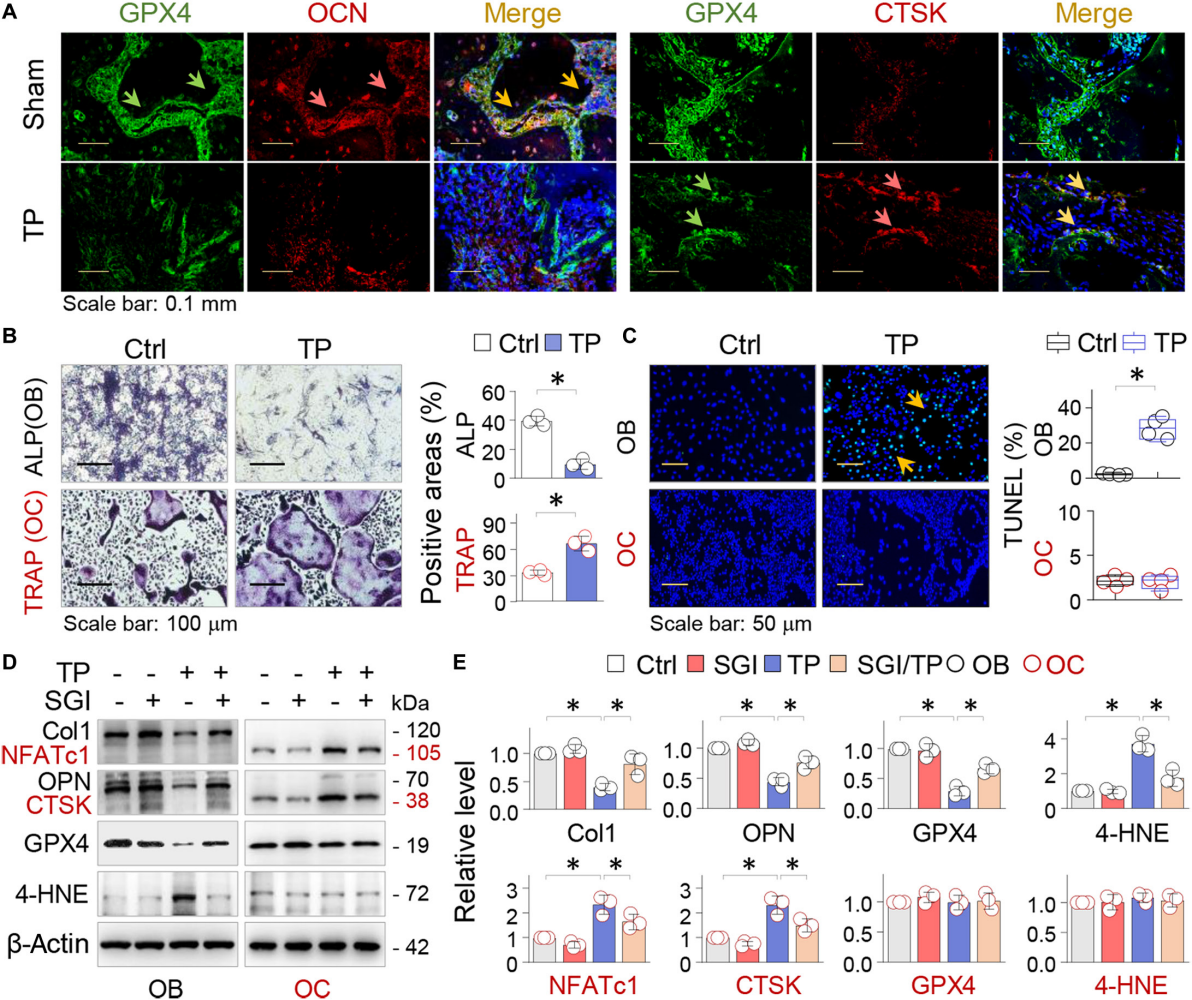
TP-induced GPX4 repression and ferroptotic alterations occur primarily in OBs. (A) Immunofluorescent double staining. Representative calvarial sections from Sham or TP-treated mice were dual-stained for GPX4 (green) with either OCN or CTSK (red), counterstained with DAPI (blue), and then merged. The double-positive cells were indicated by arrows (yellow). (B) Primary OB and OC were treated with control (Ctrl) or TP (100 μg/ml) for 7 or 5 days, respectively. Representative images of OBs and OCs stained for ALP or TRAP activities. Quantifications were presented as average ratio of positively stained area ± SD of 3 independent assays; *P < 0.05, Student’s *t* test. (C) Representative images of OB and OC treated with control or TP (100 μg/ml) for 48 h and assayed by TUNEL staining. Quantifications were presented as box-and-whisker plots (*n* = 4). *P < 0.05, Student’s *t* test. (D) OB and OC treated with control (Ctrl) or TP (100 μg/ml) in the presence or absence of SGI-1027 (10 μM) for 48 h were examined by Western blotting for Col1, OPN, NFATc1, CTSK, GPX4, and 4-HNE, with β-actin serving as control. (E) Quantifications of (D) were presented as mean ± SD of 3 separate experiments. *P < 0.05, 2-way ANOVA.

To clarify the specific cell types affected by GPX4 repression and ferroptosis, we carried out immunofluorescent double-staining on calvarial sections and observed that GPX4 was predominantly expressed in osteocalcin (OCN)-positive OBs but was significantly reduced after TP treatments, along with a concomitant decrease in OCN levels. In contrast, CTSK-positive OCs were barely detectable in the normal calvaria but increased after TP treatment, with high GPX4 expression colocalizing with CTSK-positive OCs (Fig. 5A).

Subsequently, we cultured primary OBs and OCs. TP exposure resulted in decreased ALP (alkaline phosphatase) activity in primary OBs, while it increased TRAP (tartrate-resistant acid phosphatase) activities in cultured primary OCs differentiated from bone marrow monocytes (BMMs) (Fig. 5B). However, TP treatment induced TUNEL-positive cells in OBs, not in OCs (Fig. 5C). Further experiments showed that TP treatment reduced OB markers Col1 and OPN and increased OC markers NFATc1 and CTSK. These effects were mitigated by SGI-1027. Notably, TP treatment specifically suppressed GPX4 and increased 4-HNE in OBs, which was abrogated by SGI-1027. However, in OCs, GPX4 levels and 4-HNE were not significantly affected by TP treatment (Fig. 5D and E). These findings demonstrate that GPX4 repression and ferroptotic alterations caused by DNMT aberrations occur mainly in OBs.

### GPX4 inactivation by RSL3 blocks the anti-ferroptotic effects of SGI-1027 in OBs

**Fig. 6.**
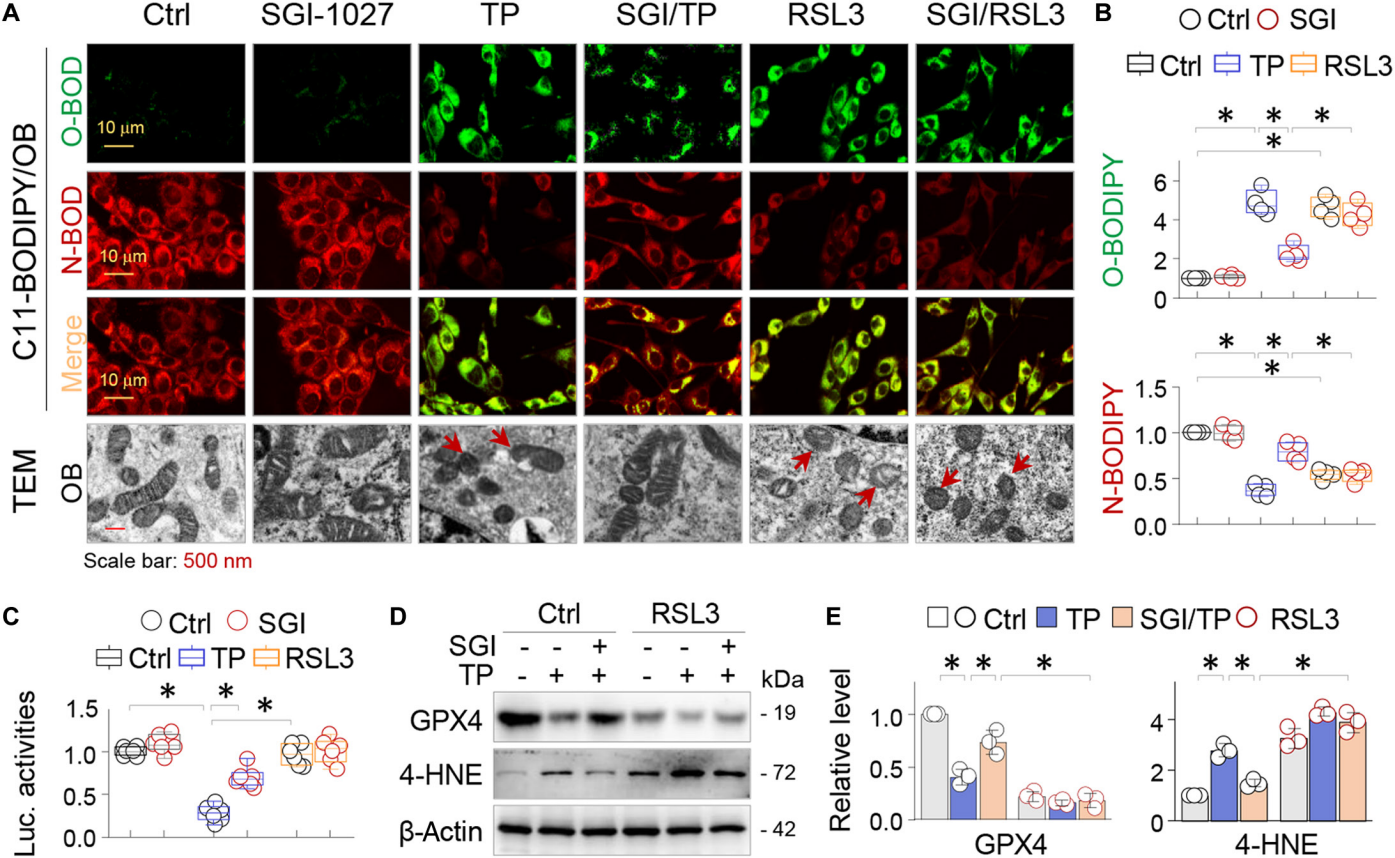
GPX4 inactivation by RSL3 blocks the anti-ferroptotic effects of SGI-1027 in OBs. (A) Representative images of OBs (48 h) treated with TP (100 μg/ml) or RSL3 (0.3 μM) either alone or in conjunction with SGI-1027 (10 μM) and then assayed by C11-BODIPY (upper 3 panels). OBs treated as above were examined by TEM. The ferroptotic mitochondrial alterations were indicated by arrows (lower panel). (B) Quantifications of N-BODIPY and O-BODIPY. The data were presented by box-and-whisker plots (*n* = 4). *P < 0.05, 2-way ANOVA. (C) Luciferase assay. A mouse *Gpx4* promoter luciferase reporter *Gpx4*p-Luc and a Renilla luciferase control were transfected into primary OBs. These cells were subsequently treated with TP (100 μg/ml), with or without SGI-1027 (10 μM) or RSL3 (0.3 μM) for 48 h. The luciferase activity of the *Gpx4* promoter was adjusted with the Renilla luciferase readings and presented by box-and-whisker plots of 6 independent assays. *P < 0.05, 2-way ANOVA. (D) Western blotting. OBs that had undergone 48-h treatments with TP (100 μg/ml) and/or SGI-1027 (10 μM) in the absence or presence of RSL3 (0.3 μM) were detected for 4-HNE and GPX4. (E) Quantification of (D). Data were presented as mean ± SD; *n* = 3; *P < 0.05, 2-way ANOVA.

To confirm the ferroptosis phenomenon of OB, we treated primary OB with TP or RSL3 (a compound that inactivates and degrades GPX4) [25] in the absence or presence of SGI-1027, followed by treatment with C11-BODIPY (boron dipyrromethane difluoride), a fluorescent lipid oxidation sensor. Control OBs showed predominantly non-oxidized BODIPY (N-BODIPY) and hardly noticeable oxidized BODIPY (O-BODIPY). However, TP or RSL3 treatment reversed the N-BODIPY/O-BODIPY distribution (Fig. 6A, top 3 panels, and B). Furthermore, both TP and RSL3 caused ferroptotic mitochondria changes such as small mitochondria and diminished mitochondria crista, as observed by TEM (Fig. 6A, lower panel). However, SGI-1027 effectively reversed N-BODIPY/O-BODIPY distribution and normalized the mitochondrial changes induced by TP but not those caused by RSL3 (Fig. 6A and B), indicating that SGI-1027 protects OBs from TP-induced ferroptosis via reinstating GPX4.

We next transfected a *Gpx4* promoter luciferase reporter plasmid into OBs and observed that transcription repression of *Gpx4* promoter caused by TP was restored by SGI-1027. However, RSL3 had no effect on GPX4 transcription (Fig. 6C). Finally, SGI-1027 corrected TP-induced suppression of GPX4 protein and 4-HNE induction in OBs, but these effects were abolished by RSL3 (Fig. 6D and E). These results indicate that OB ferroptosis is mainly caused by DNA methylation-induced GPX4 transcriptional suppression.

### Osteoblastic GPX4 haplodeficiency exacerbates TP-induced osteolysis

**Fig. 7.**
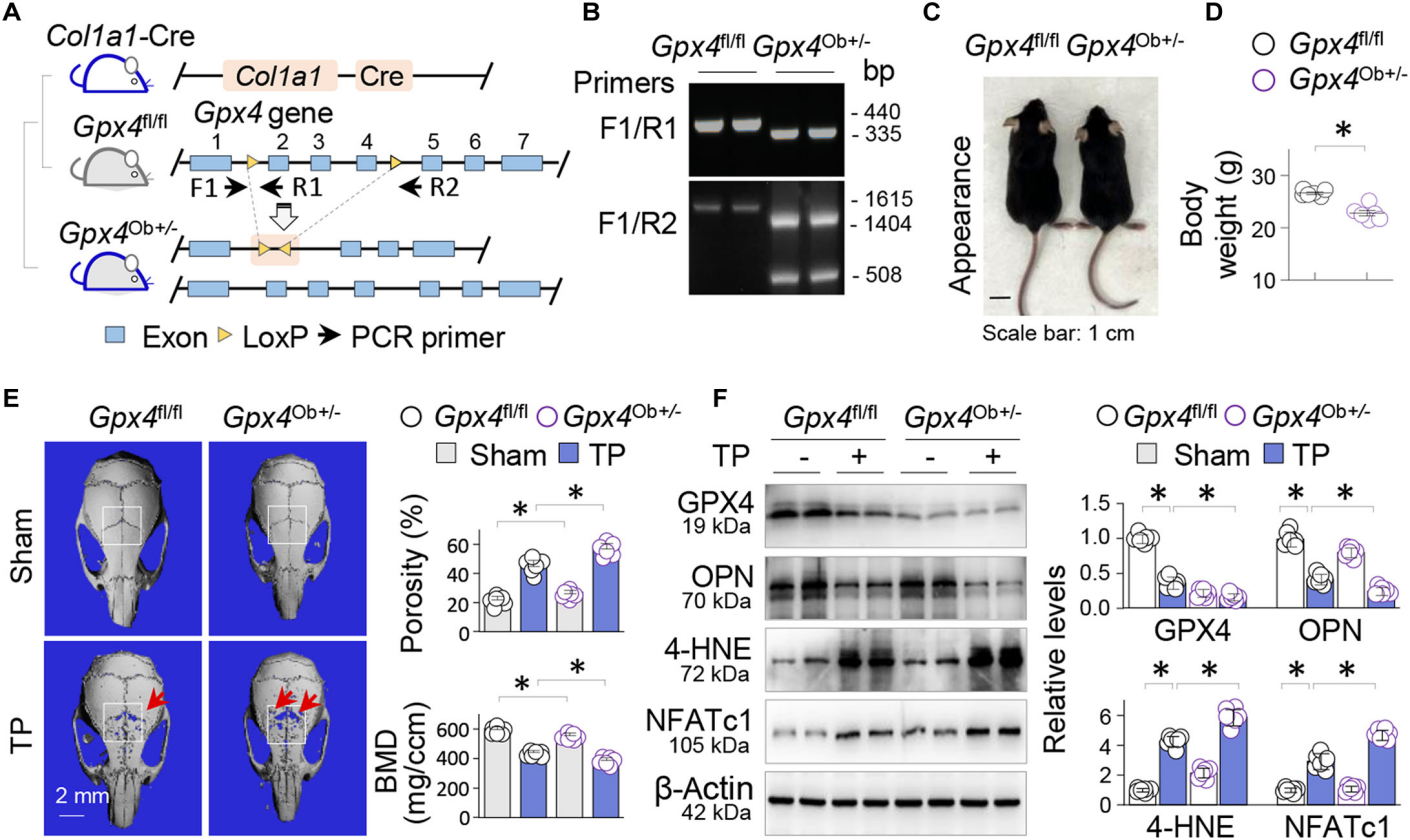
Osteoblastic GPX4 haplodeficiency exacerbates TP-induced osteolysis. (A) Schematic generation of OB-specific *Gpx4* haplodeficient mice *Gpx4*^Ob+/−^ by crossing *Gpx4*-flox mice (*Gpx4*^fl/fl^) with transgenic *Col1a1*-Cre mice. (B) Genotyping of *Gpx4*^fl/fl^ and *Gpx4*^Ob+/−^ mice by PCR with primers F1/R1 and R1/R2 and PCR product analysis on agarose gels. The sizes (bp) of PCR products were indicated. (C) Appearance and (D) quantification of body weight of *Gpx4*^fl/fl^ and *Gpx4*^Ob+/−^ mice at 8 weeks; *n* = 6; *P < 0.05, Student’s *t* test. (E) Representative 3D μCT calvarial images of *Gpx4*^fl/fl^ and *Gpx4*^Ob+/−^ mice. The ROIs were indicated by squares, while the areas of resorption were highlighted by arrows. Quantitative analyses of bone porosity and BMD were presented as mean ± SEM. *P < 0.05, 2-way ANOVA. (F) Western blotting of calvarial tissues for GPX4, 4-HNE, NFATc1, and OPN, with β-actin serving as control. Two random samples from each group were shown. Quantitative analysis was presented as mean ± SEM; *n* = 6; *P < 0.05, 2-way ANOVA.

To further verify the impact of osteoblastic GPX4 repression on osteolytic bone damage, we intended to generate a mouse strain with OB *Gpx4* knockout by crossing *Col1a1-Cre* transgenic mice with *Gpx4*-floxed mice (*Gpx4*^fl/fl^), in which the exons 2 and 4 of the mouse *Gpx4* gene were flanked by loxP sites (Fig. 7A). However, we only obtained *Gpx4* wild-type (*Gpx4*^fl/fl^) or *Gpx4* haplodeficient mice *Gpx4*^Ob+/−^. Genotyping confirmed that all surviving *Gpx4*-deficient mice were heterozygous (Fig. 7B), indicating that homozygous *Gpx4* knockout in OBs is also lethal.

Compared to *Gpx4*^fl/fl^ mice, *Gpx4*^Ob+/−^ mice at 8 weeks appeared smaller, had a lighter body weight (Fig. 7C and D), and exhibited reduced BMD and increased calvarial porosity before (27.4 ± 1.1% in *Gpx4*^Ob+/−^ versus 23.1 ± 1.4% in *Gpx4*^fl/fl^ mice, *P* < 0.05; Fig. 7E) and after TP treatment (58.3 ± 1.9% in *Gpx4*^Ob+/−^ versus 47.1 ± 2.1% in *Gpx4*^fl/fl^ mice, *P* < 0.05; Fig. 7E). Furthermore, TP treatment aggregated the deleterious expression of osteoclastic NFATc1, osteoblastic OPN, and 4-HNE in *Gpx4*^Ob+/−^ mice (Fig. 7F). These findings indicate that proper levels of osteoblastic GPX4 is crucial for maintaining bone structural integration and that even osteoblastic GPX4 haplodeficiency exacerbates TP-induced ferroptotic osteolysis.

### Osteoblastic GPX4 is critical for the anti-ferroptotic and osteoprotective effects of SGI-1027 in vivo

**Fig. 8.**
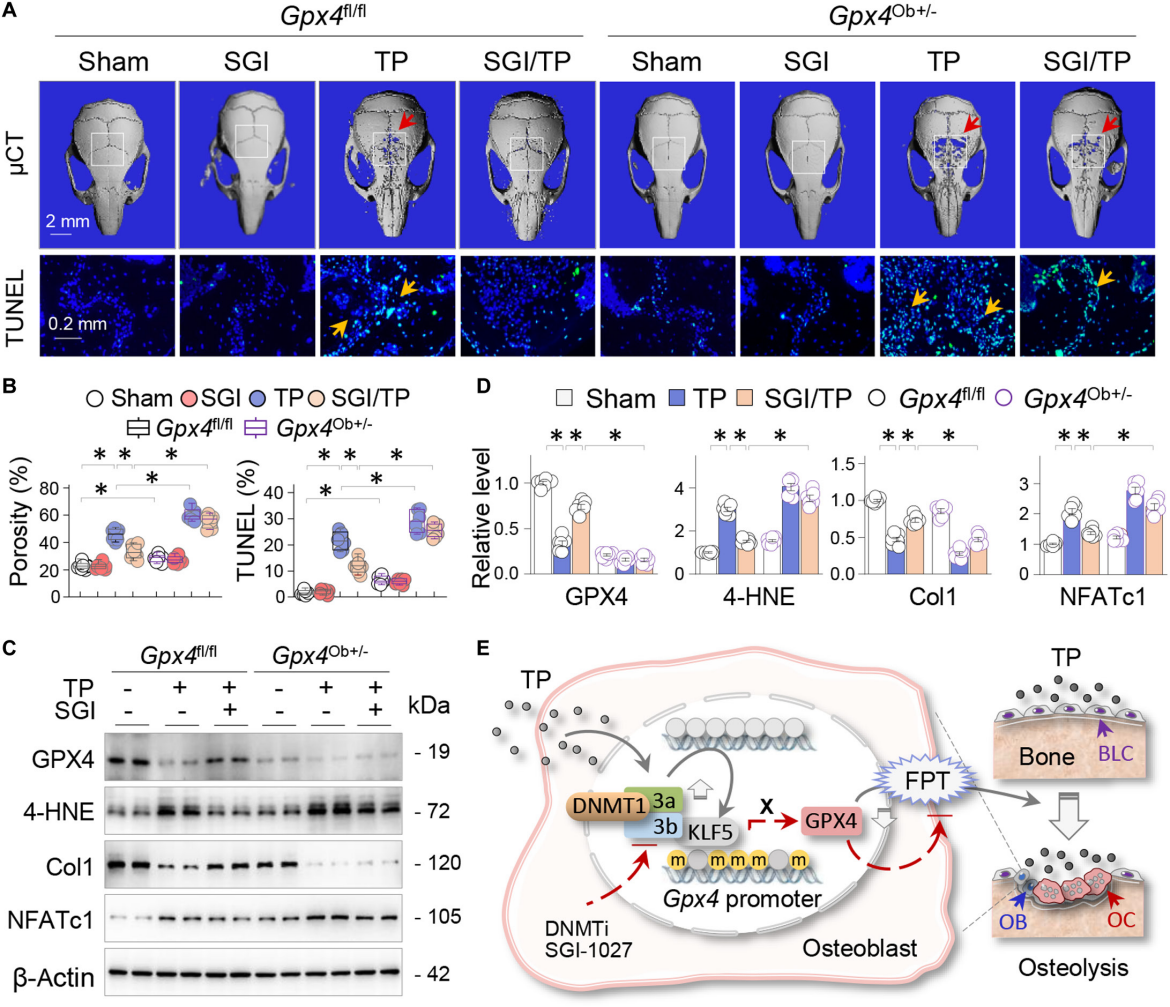
OB GPX4 is critical for the anti-ferroptotic and osteoprotective effects of SGI-1027 in vivo*. Gpx4*^fl/fl^ and *Gpx4*^Ob+/−^ mice were divided into Sham, SGI-1027, TP, and SGI-1027/TP groups (*n* = 6, 2 weeks). (A) Representative images of 3D μCT calvaria (upper panel) and TUNEL-stained calvarial sections (lower panel). The ROIs of μCT images were indicated by squares, while the resorption areas and TUNEL-positive cells were indicated by arrows. (B) Quantification of (A). Data were presented as box-and-whisker plots (*n* = 6); *P < 0.05, 3-way ANOVA followed by Tukey’s post hoc test. (C) Western blotting of calvarial tissues for GPX4, 4-HNE, Col1, and NFATc1, with β-actin serving as control. Two random samples per group were presented. (D) Quantification of (C). Data were depicted as mean ± SEM; *P < 0.05, 2-way ANOVA. (E) Schematic diagram of sequential TP treatment, DNMT1/3a/3b elevations, GPX4 promoter hypermethylation (m), KLF5-assisted GPX4 transcriptional suppression, and resulting OB ferroptosis (FPT) that promote osteolysis. Contrarily, DNMT inhibition (DNMTi) by SGI-1027 blocks the processes (BLC, bone lining cell).

To verify the essential role of GPX4 restoration by DNMT inhibition in ferroptotic osteolysis in vivo, we compared the osteoprotective effects of SGI-1027 between *Gpx4*^fl/fl^ and *Gpx4*^Ob+/−^ mice. As expected, *Gpx4*^Ob+/−^ mice exhibited increased calvarial porosity (Fig. 8A, upper panel, and B) and TUNEL-positive cells (29.1 ± 1.7% in *Gpx4*^Ob+/−^ versus 21.5 ± 1.4% in *Gpx4*^fl/fl^ mice, *P* < 0.05; Fig. 8A, lower panel, and B) before and after TP treatment. Administration of SGI-1027 effectively reduced TP-induced porosity changes (33.3 ± 2.1% in SGI-1027/TP versus 45.9 ± 1.8% in TP, *P* < 0.05) and TUNEL-positive cell counts (11.9 ± 1.1% in SGI-1027/TP versus 21.5 ± 1.4% in TP, *P* < 0.05) in *Gpx4* ^fl/fl^ mice; however, the beneficial effects were significantly reduced in *Gpx4*^Ob+/−^ mice (Fig. 8A and B). Comparably, SGI-1027 significantly normalized TP-induced adverse expression of GPX4, 4-HNE, Col1, and NFATc1 in *Gpx4*^fl/fl^ mice, but these effects were largely abolished in *Gpx4*^Ob+/−^ mice (Fig. 8C and D). Overall, these findings strongly suggest that osteoblastic GPX4 restoration by SGI-1027 is pivotal for its anti-ferroptosis and osteoprotective functions.

## Discussion

In this study, we obtained normative data to facilitate a deeper comprehension of the pathogenesis of ferroptosis and osteolysis caused by TP. We found that TP-induced ferroptosis and osteolysis were accompanied by transcriptional repression of the key anti-ferroptosis factor GPX4, aberrant DNMT1/3a/3b elevations, and hypermethylation of the *Gpx4* promoter and were transcriptionally promoted by KLF5 and NCoR. On the other hand, DNMT inhibition by SGI-1027 efficiently reversed the hypermethylation of the *Gpx4* promoter and the resulting GPX4 repression, and alleviated ferroptosis and osteolytic bone pathologies. Furthermore, we demonstrated that ferroptosis occurred primarily in OBs and generated a strain of mice with haploinsufficiency of *Gpx4* specifically in OBs (*Gpx4*^Ob+/−^), which developed worse ferroptotic osteolysis before and after TP treatments and blunted the anti-ferroptosis and osteoprotective effects of DNMT inhibition by SGI-1027 (Fig. 8E). Thus, our study has identified a key epigenetic pathway of OB ferroptosis that is crucial for TP-induced osteolysis.

The identification of aberrant DNMT1/3a/3b elevation as a causative epigenetic event of ferroptotic *Gpx4* repression, which is critically involved in TP-induced osteolysis, is a notable achievement of our study. Recent studies report that GPX4 repression contributes significantly to ferroptosis in metal particle-induced osteolysis [13–15], but these studies lacked mechanistic insights into the precise nature of repression. We found that ferroptotic GPX4 repression coincided with DNMT1/3a/3b aberration and hypermethylation of the GPX4 promoter (Fig. 2). The methylation level of a given gene promoter or enhancer is positively regulated by DNMTs and methyl donors [29], and negatively influenced by DNA insulators [30] and DNA demethylating TET enzymes [31]. We also demonstrated that the DNMT inhibitor SGI-1027 effectively reversed GPX4 epigenetic repression and associated ferroptotic osteolysis (Figs. 2 and 4), indicating that the pathological DNMT1/3a/3b increases cause GPX4 repression. To our knowledge, this is the first report to identify the upstream regulators of DNA methylation-associated ferroptosis that are critically involved in metal particle-induced osteolysis.

Epigenetic DNA methylation modifications might affect many components of ferroptotic signaling pathways. For example, several ferroptosis-related genes, including *FSP1*, *CDH1*, *SLC7A11*, and *SLCA2*, are likely targets of DNMT modifications [32]. As a pivotal enzyme, GPX4 directly restrains the accumulation of lipid peroxides, safeguarding cells against ferroptotic damage. Numerous studies have confirmed that correcting GPX4 transcriptional suppression with pharmacological agents effectively blocks ferroptosis in various cell types. For example, in the mouse brain of Alzheimer’s disease model, neuronal GPX4 suppression and ferroptosis are accompanied by repression of peroxisome proliferative-activated receptor-α (PPAR-α), while the PPAR-α agonist GW7647 effectively reverses GPX4 transcriptional suppression and improves brain ferroptotic injuries [33]. Additionally, in mouse kidneys with acute kidney injury (AKI) induced by aristolochic acid I or folic acid, marked tubule ferroptosis with GPX4 transcriptional suppression and preferential histone deacetylase HDAC3 elevation occurs; however, the HDAC3 inhibitor RGFP966 GPX4 restoration-sensitively alleviates the ferroptotic pathologies [34]. In this study, we further demonstrated that *Gpx4*-deficient mice developed worse ferroptotic osteolysis before and after TP treatment, which was effectively mitigated by SGI-1027 in a GPX4-dependent manner (Figs. 7 and 8). This indicates that GPX4 is a key mediator of DNA methylation aberration-incurred ferroptosis and an effective target of DNMT intervention.

The discovery that KLF5 is actively involved in *Gpx4* transcriptional suppression caused by DNA methylation is an intriguing observation from our study. DNA methylation is known to silence gene transcription through a repressive complex that includes various transcriptional cofactors, such as DNMTs, DNA methylation binding proteins, transcriptional regulators/co-repressors, and histone deacetylases [35]. KLF5 has a dual function on transcription of the target gene. For instance, KLF5 up-regulates the pro-apoptotic gene *BAX* following ultraviolet irradiation [36] and represses 2 major pro-inflammatory cytokines (*Il1b* and *Tnfa*) under ischemic–reperfusion condition [27]. We observed increased KLF5 in bone tissues after TP treatment along with NCoR (but not the up-regulated SMRT or SnoN), bound inducibly to the KLF5 motif region located in the proximal promoter of *Gpx4*, which was inhibited by DNMT inhibition (Fig. 3B and C). Furthermore, GPX4 repression was sensitive to KLF5 inhibition by ML264 (Fig. 3D and E). These data indicate that KLF5 functions as a vital co-regulatory factor in the repression of *Gpx4* transcription caused by DNMT aberration during TP-incurred osteolytic ferroptosis.

In recent years, studies have shown that various cells in bone tissue could undergo ferroptosis under different pathological conditions, including osteocytes [37], mesenchymal stem cells [38], OBs, and OCs [15,39,40]. More pertinently, it is well established that OBs and OCs respond oppositely to metal particle-induced oxidative stress, which stimulates osteoclastogenesis [41] but inhibits osteoblastogenesis [42]. Accordingly, OBs, but not OCs, are sensitive to regulated cell death and oxidative damage, leading to osteolysis [4,43]. In this study, we demonstrated that the DNMT aberration-induced GPX4 repression and subsequent ferroptotic changes occurred mainly in OBs (Figs. 5 and 6) rather than OCs, and that osteoblastic *Gpx4* haplo-knockout worsened TP-induced ferroptotic and osteolytic damage. This supports the vital role of osteoblastic ferroptosis in metal particle-induced osteolysis. Furthermore, the precise mechanisms leading to differential GPX4 repression and ferroptosis in OBs versus OCs are currently unclear, but epigenetic modifications are specific in different tissues, cells, and even individual genes. This suggests that epigenetic GPX4 suppression might be a bifurcation point that differentiates OBs from OCs in response to ferroptotic insults.

In conclusion, we have discovered that increases in DNMT1/3a/3b and the resulting GPX4 repression play a causal role in TP-induced ferroptotic osteolysis, which is effectively alleviated by the DNMT inhibitor SGI-1027. Various pharmacological inhibitors, such as liproxstatins and ferrostains, have been used to study the function and regulation of ferroptosis, but their use in vivo is limited due to low stability and efficacy [44]. Given that epigenetic changes are theoretically reversible and DNA demethylating drugs have been approved for the clinical treatment of acute myeloid leukemia and myelodysplastic syndromes [45], our study not only uncovers the epigenetic characteristics of TP-induced ferroptotic osteolysis but also proposes a feasible intervention strategy that could benefit patients with metal prosthesis complications.

## Materials and Methods

### Animal study

C57BL/6J mice were obtained from Gempharmatech Co. (Nanjing, China). Generation of OB-specific *Gpx4*-deficient mice was intended by crossing *Gpx4*-floxed mice (*Gpx4*^fl/fl^, Gempharmatech Co., China, strain no. T050827) with transgenic *Col1a1-Cre* mice (*Col1a1*-Cre, Gempharmatech Co., China, strain no. T004734). Mouse genotyping was performed with primers F1: GTACTGCAACAGCTCCGAGTTC; R1: ACTTATCCAGGCAGACCATGTG; R2: AACTCCAATTCCCAGGACTCAC. However, only wild-type and *Gpx4* haplodeficient mice *Gpx4*^Ob+/−^ survived to birth (Fig. 7A to C), suggesting that homozygous *Gpx4* knockout in OBs is embryonically lethal. All mice were housed in a standardized specific pathogen-free environment and provided with adequate chow and water. All animal procedures were approved by the Ethics Committee and the Institutional Animal Care and Use Committee of Nanjing Drum Tower Hospital (20201220) and complied with ARRIVE (Animal Research: Reporting of In Vivo Experiments) guidelines [46].

For intervention study, male C57BL/6J mice of 8 to 10 weeks were randomly divided into 4 groups with 6 mice in each group: (a) Sham vehicle control (40 μl of sterile phosphate-buffered saline (PBS) was implanted beneath the periosteum at the site of skull suture); (b) SGI-1027 or Lip-1: daily one intraperitoneal injection of SGI-1027 (2.5 mg/kg) [47] or Lip-1 (10 mg/kg) [25], both from MCE, New Jersey, USA, dissolved in 2% dimethyl sulfoxide (DMSO), 2% Tween 80, and 30% polyethylene glycol (PEG) 300; (c) mouse model of calvarial osteolysis: TP (20 mg in 40 μl of sterile PBS, 7440-32-6, Alfa Aesar, Thermo Fisher Scientific Inc., UK) was implanted beneath the periosteum at the skull suture after isoflurane anesthesia [1,8,24]; (d) SGI-1027 or Lip-1 intervention. SGI-1027 or Lip-1 was administered 1 day after TP treatment. In experiments to determine the role of GPX4 in TP-induced ferroptotic osteolysis and SGI-1027 intervention, *Gpx4*^fl/fl^ and *Gpx4*^Ob+/−^ mice were subjected to the same treatments above. Two weeks later, experimental mice were sacrificed and calvaria tissues were collected for further analyses.

### Human samples

Human bone tissue samples were obtained from patients who underwent hip replacement surgery at Nanjing Drum Tower Hospital of Nanjing University Medical School. PPO tissues were collected from 6 patients (61 to 73 years old) who developed severe prosthesis loosening after hip replacement. Control tissues were from 6 patients diagnosed of femoral neck fracture and receiving hip joint replacement for the first time. The study was approved by Human Research Ethics Committee of Nanjing Drum Tower Hospital (2020-156-01), and all participants provided written informed consents. Patients are not involved in the design, conduct, reporting, or dissemination plans of the research.

### Micro-x-ray computed tomography analysis

Calvarial micro-architectures were analyzed by a microCT scanner (VivaCT80; Scanco Medical AG, Switzerland) with scanning parameter setting at 70 kV, 114 μA, and a spatial resolution of 15.6 μm per pixel. A square region of interest (ROI) centered on the midline suture was demarcated, and the osteoporosity and BMD in ROI were calculated using SkyScan software. Three-dimensional (3D) calvarial images were reconstructed utilizing Scanco Medical AG software.

### H&E, Perl’s prussian blue, IHC, immunofluorescent, and TUNEL staining

Calvarial coronal sections of 5 μm thickness were prepared as before [24] and stained by H&E (hematoxylin and eosin) or immunohistochemistry (IHC) for GPX4 (ab231174, Abcam, Cambridge, UK), DNMT1 (D63A6, Cell Signaling Technology, Boston, USA), DNMT3a (ab188470, Abcam, Cambridge, UK), and DNMT3b (AF300068, AiFangbiological, Changsha, China), following routine protocols [48,49]. Perls’ prussian blue–DAB (3,3′-diaminobenzidine) staining (G1428, Solarbio, Beijing, China) and TUNEL (G1504-50T, Servicebio, China) staining followed the manufacturer’s protocols.

Immunofluorescent double staining was carried out as previously described [24]. Initially, calvarial sections were incubated with primary antibody rabbit anti-GPX4 (A1933, ABclonal, Wuhan, China) plus mouse anti-CTSK (37259, Abcam, Cambridge, UK) or mouse anti-OCN (sc-365797, Santa Cruz Biotechnology, USA) overnight and then treated with secondary antibodies 594-conjugated goat anti-mouse immunoglobulin G (IgG) (H+L) (AS054) and 488-conjugated goat anti-rabbit IgG (H+L) (AS053, ABclonal, China), with 4′,6-diamidino-2-phenylindole (DAPI) (S2110, Solarbio, Beijing, China) being used for nuclear staining. Finally, fluorescence Images were captured by a laser confocal microscope (FV3000, Olympus, Tokyo, Japan).

### TEM examination

Fresh mouse calvaria were promptly washed with PBS and immersed in a fixative solution composed of 2% paraformaldehyde and 2.5% glutaraldehyde. Adhering to a standard TEM sample preparation routine, the specimens underwent sequential rinses, fixation in 1% osmium tetroxide, dehydration, and infiltration with Epon812 resin. Thereafter, ultrathin sections were stained with citrate lead and uranyl acetate, and inspected utilizing a JEOL-1200EX microscope.

### Primary cell culture

Primary OBs were isolated and cultured following a previously established method [24]. The procedure involved removing the periosteum from newborn mouse skulls, subjecting them to 3 sequential digestions with 0.1% collagenase A (4155743, Roche, Basel, Switzerland), collecting the final cell pellets, and culturing in α-MEM (minimum essential medium) media containing 10% fetal bovine serum (FBS) (085-150, WISENT, Nanjing, China) and 50 μg/ml ascorbic acid (S3114, Selleck Chemicals lnc., TX, USA). Only cells prior to passage P2 were used for in vitro experiments.

Primary OCs were isolated from BMMs of femur of 4-week-old male mice following a protocol essentially as before [24]. The isolated cells were cultured in α-MEM media [containing 10% FBS plus 30 ng/ml macrophage colony-stimulating factor (M-CSF) (CB34, Novoprotein, Shanghai, China)]. After a span of 3 days, additional 50 ng/ml RANKL (C28A, Novoprotein, Shanghai, China) was added to facilitate OC differentiation before assays.

### Cell staining for TRAP and ALP activities

OC TRAP activity staining was performed with a commercial kit (387A-1KT, Sigma-Aldrich, USA) following standard instruction. The differentiated OCs treated with TP (100 μg/ml) for 5 days were performed before assay. OB ALP (alkaline phosphatase) activity staining was carried out with BCIP/NBT Alkaline Phosphatase Color Development Kit (KGE1111-100, KeyGEN BIOTECH, Nanjing, China). The cultured primary OBs treated with TP (100 μg/ml) for 7 days were assayed for ALP activities [50]. Five pre-determined locations with positive staining were quantitatively analyzed using ImageJ software.

### C11-BODIPY staining

C11-BODIPY is a lipid oxidation sensor, with the excitation and emission wavelengths of 581/591 nm for reduced, while 488/510 nm for oxidized, probes [51]. We carried out the assay with C11-BODIPY (RM02821, ABclonal, Wuhan, China) following the manufacturer’s protocol. In brief, OBs subjected to different treatments were exposed to C11-BODIPY dye for 30 min and then observed under a confocal fluorescence microscopy. The average fluorescent intensities of oxidized and non-oxidized BODIPY for each treatment condition were calculated from 5 randomly selected fields and adjusted by cell numbers using ImageJ software.

### Western blotting

Western blotting assays of mouse calvarial or cell were performed as before [48,52]. Primary antibodies used were as follows: DNMT3b (AF300068, 1:1,000) and NFATc1(AF06823, 1:2,000) from AiFangbiological, Changsha, China; GPX4 (A1933, 1:1,000) and β-actin (AC026, 1:100,000) from ABclonal, Wuhan, China; Col1 (GB114197, 1:800) and OPN (GB112328, 1:2,000) from Servicebio, Wuhan, China; MDA (abx445120, Abbexa, Cambridge, UK, 1:1,000); 4-HNE (ab46545, Abcam, Cambridge, UK, 1:1,000); CTSK (DF6614, 1:1,000), KLF5 (AF7542, 1:1,000), SnoN (DF3088, 1:1,000), NCoR (AF0270, 1:1,000), and SMRT (DF8896, 1:1,000) from Affinity Biosciences, Changzhou, China. The horseradish peroxidase (HRP)-conjugated secondary antibodies were obtained from Proteintech, Wuhan, China. Western blots were visualized with NcmECL Ultra (P10300, New Cell & Molecular Biotech, Suzhou, China) plus Western blotting detection system (Tanon, Shanghai, China), and the protein levels were quantified using ImageJ software.

### Methylated specific PCR (MSP) and bisulfite-sequencing PCR (BSP)

We used online MetPrimer software (http://www.urogene.org/methprimer) [53] to analyze *Gpx4* promoter for identification of CpG islands and primer designing of MSP and BSP. Total DNA extraction was carried out employing a DNeasy Blood & Tissue Kit (69504, QIAGEN, Dusseldorf, Germany), whereas the DNA Bisulfite Conversion Kit (DP215, TIANGEN Biotech, Beijing, China) was utilized to convert unmethylated cytosine residues into uracil, adhering strictly to the manufacturer’s guidelines. MSP and BSP procedures were executed in accordance with previously documented methodologies [49].

For MSP assay of mouse *Gpx4* promoter, the primers used for MSP assay of mouse *Gpx4* promoter were as follows: methylated forward primer 5′-TTTTTTAAGGGGATGATTTTGATAC (located at −247/−223) and reverse primer 5′-ATACCCAATAATAAAAACGCGAA (located at −78/−100); unmethylated forward primer 5′-TTTTAAGGGGATGATTTTGATATGT (located at −245/−221) and reverse primer 5′-CATACCCAATAATAAAAACACAAA (located at −77/−100). The input PCR used forward primer 5′-CTCTTTAAGGGGATGACTTTGACAC and reverse primer 5′-ATGCCCAGTGATAGGGACGCGGG. PCR products were electrophoresed on 1.5% agarose gels, and the densitometry analyses were performed with ImageJ. The intensity of each PCR product was initially normalized with input product, followed by the computation of the percentage of methylated and unmethylated PCR amplicons in relation to the overall PCR products.

BSP was conducted on 3 mice randomly selected from each group to examine the methylation patterns and precise locations within the *Gpx4* promoter according to a previously reported methodology [54]. Briefly, the genomic DNAs of calvarial homogenates were treated with bisulfite and PCR-amplified with *Gpx4* forward primer 5′-GTTTTTTAAGGGGATGATTTTGATA (−248/−224) and *Gpx4* reverse primer 5′-CCCTACAACCAATAAAAAACCTAAATA (5/−22). The PCR products were run on a 2% agarose gel, subsequently purified utilizing a Nucleotide Purification Kit (B518131, Sangon Biotech, China), and then inserted into pGEM-TEasy-vector (A1380, Promega, Madison, USA). From every PCR sample, 5 bacterial colonies were arbitrarily picked and plasmid DNA was extracted for sequencing. The proportions of methylated CpGs over total CpGs within the cloned fragments were determined.

### Reverse transcription PCR and quantitative real-time PCR

Reverse transcription PCR (RT-PCR) was performed as before [55]. In brief, calvarial tissue-derived total RNA was extracted and reversely transcribed to cDNA using HiScript Q RT SuperMix for qPCR (R122-01, Vazyme Biotech, China). RT-PCR was processed with Taq Plus Master Mix (P212-01, Vazyme Biotech, China) followed by 1% agarose gel electrophoresis, and quantitative real-time PCR (qRT-PCR) was carried out with ChamQ Universal SYBR qPCR Master Mix (Q711-02, Vazyme Biotech, China) on a quantitative real-time PCR instrument (Thermo Fisher Scientific, USA). PCR was performed with mouse *Gpx4* forward primer 5′-TGTGCATCCCGCGATGATT and reverse primer 5′-CCCTGTACTTATCCAGGCAGA; mouse *Gapdh* forward primer 5′-TGGATTTGGACGCATTGGTC and reverse primer 5′-TTTGCACTGGTACGTGTTGAT were used as PCR internal control. Human *GPX4* forward primer 5′-GAGGCAAGACCGAAGTAAACTAC and reverse primer 5′-CCGAACTGGTTACACGGGAA and human *GAPDH* forward primer 5′-GGAGCGAGATCCCTCCAAAAT and reverse primer 5′-GGCTGTTGTCATACTTCTCATGG were used as PCR internal control. For qRT-PCR analysis, expression levels were quantified employing the 2^−ΔΔCt^ formula and represented as the relative fold differences.

### Chromatin immunoprecipitation

ChIP assay of calvarial tissues was carried out with the same antibodies in Western blotting to KLF5, NCoR , SMRT, or SnoN, essentially as before [55]. The initial (input) and immunoprecipitated DNA fractions underwent additional amplification via RT-PCR with primers for the *Gpx4* promoter (forward, 5′-GGGGATGACTTTGACACGC and reverse*,* 5′-GCCTGAATGAAGGGACGG, which encompass the −239 to −14 locus including a predicted binding motif for KLF5 (−43/gccccgccca). The RT-PCR products were electrophoresed on 1.5 % agarose gels and visualized under ultraviolet light.

### Luciferase assay

Primary OBs were transfected with a *Gpx4* promoter reporter plasmid *Gpx4*p-luc [34] and a renilla luciferase reporter plasmid as control. After various treatments with TP, SGI-1027, ML264, or RSL3 as indicated, luciferase activities were measured using the Dual-Luciferase Reporter Assay System (E1910, Promega, USA). The luciferase activity of the *Gpx4* reporter was adjusted with those of renilla’s and presented as relative fold differences.

### Statistical analysis

We assessed the data normal distributions and the assumptions of homogeneity of variances by Shapiro–Wilk test and Levene’s test, respectively. We used GraphPad Prism to analyze data and plot quantitation graphs. Effect size (large effect size, η^2^ ≥ 0.1379; medium effect size, 0.0588 ≤ η^2^ < 0.1379; small effect size, 0.0099 ≤ η^2^ < 0.0588) [56] was calculated using SPSS V22.0 software. Data from animal studies and cell assays are presented as mean ± SEM or mean ± SD, respectively. Box-and-whisker plot is defined as the central line for median, the bounding box for the interquartile range from 25th to 75th percentiles, and the whiskers for the minimum and maximum. Student’s *t* test was used for 2-group comparison, 2-way analysis of variance (ANOVA) for 2-factor experiment, and 3-way ANOVA followed by Tukey’s post hoc test for multiple group comparison. Statistical significance is set at *P* < 0.05.

## Data Availability

Data are available from corresponding authors upon reasonable request.
